# “It doesn’t seem to make sense for a company that sells cigarettes to help smokers stop using them”: A case study of Philip Morris’s involvement in smoking cessation

**DOI:** 10.1371/journal.pone.0183961

**Published:** 2017-08-28

**Authors:** Patricia A. McDaniel, E. Anne Lown, Ruth E. Malone

**Affiliations:** Department of Social and Behavioral Sciences, School of Nursing, University of California, San Francisco, San Francisco, CA, United States of America; University of California, San Diego, UNITED STATES

## Abstract

**Background:**

In the late 1990s, American tobacco companies began offering limited cessation assistance to smokers by posting links on their company websites to government-sponsored smoking cessation resources. Philip Morris USA (PM) went further, funding youth cessation programs and creating its own online cessation program, QuitAssist. We explore why PM entered the cessation arena, and describe the variety of options considered and how PM-supported cessation programs were evaluated and promoted.

**Methods:**

We retrieved and analyzed archival PM documents from 1998–2005. We supplemented information from the documents with scholarly articles assessing QuitAssist and archived versions of the PM and QuitAssist websites.

**Results:**

PM’s Youth Smoking Prevention department began funding youth cessation projects and programs soon after its creation in 1998, motivated by the same issue that drove its interest in youth smoking prevention: regulatory threats posed by public and policymaker concern about youth smoking. The department took a similar approach to youth smoking cessation as it did with prevention, rejecting curricula with “anti-industry” themes. In 2002, a “cessation exploration team” examined a variety of rationales for and approaches to company support for adult smoking cessation. Ultimately, PM chose QuitAssist, a limited and less expensive option that nonetheless provided opportunities for engagement with a variety of public health and government officials. Independent research indicates that QuitAssist is not an effective cessation tool.

**Conclusions:**

While the transformation of ambitious plans into a mundane final product is a recurring theme with PM’s corporate responsibility efforts, it would be inappropriate to dismiss PM’s smoking cessation endeavors as half-hearted attempts to appear responsible. Such endeavors have the potential to inflict real harm by competing with more effective programs and by helping to maintain a tobacco-favorable policy environment. If PM truly wanted to support cessation, it could drop legal and other challenges to public policies that discourage smoking.

## Introduction

Traditionally, tobacco companies’ interest in smoking cessation was limited to undermining it [1, 2]. Beginning in the late 1990s, however, tobacco companies such as Philip Morris USA (PM), RJ Reynolds, and Lorillard began offering limited cessation assistance to smokers by posting links on their company websites to government-sponsored smoking cessation resources [3–5]. Soon after, PM began funding youth smoking cessation programs and created its own online smoking cessation program, QuitAssist, which it continues to maintain.

This move was not entirely unprecedented. Since the 1980s, tobacco companies had devoted resources to a variety of youth smoking prevention (YSP) programs. Research has shown, however, that such programs were not intended to reduce youth smoking prevalence. Instead, they served tobacco companies’ political interests by enhancing the industry’s image and credibility [6]; marginalizing public health advocates [7]; creating allies among policymakers and regulators [7]; forestalling effective tobacco control legislation and preventing enforcement of existing tobacco control laws [6, 7]; providing a litigation defense [8]; and directing funds away from programs that work (e.g., those that directly confront the tobacco industry) and toward programs in which the industry could be a partner [8].

Tobacco industry promotion of smoking *cessation*, however, is a more recent phenomenon and has been subject to less scrutiny than its YSP programs. Previous research, for example, has examined the content and relative popularity of PM’s QuitAssist website and its impact on smokers [9–12]; however, no research has explored the internal development of PM’s smoking cessation programs for youth and adults. We analyzed PM documents to understand why PM entered the cessation arena, and to describe the variety of cessation options considered and how PM-supported cessation programs were evaluated and promoted. We show that while PM’s motivation for and approach to youth smoking cessation had parallels with its motivation for and approach to YSP, it had difficulty articulating its interest in adult cessation. In addition, although PM regarded itself as uniquely qualified to create a successful adult cessation program, the final product failed to include many of the elements that the company or its consultants had identified as key to success.

## Methods

Litigation against the tobacco industry has resulted in release of more than 14 million previously undisclosed industry documents [13, 14] now archived at the University of California, San Francisco in a full-text searchable electronic repository [15]. We searched the archives using standard tobacco industry document search strategies [14, 16, 17], which include starting with broad search terms (“QuitAssist,” “smoking cessation”) and using retrieved documents to identify more specific terms, a process termed “snowball sampling.” We identified 330 PM documents from 1998–2005. We also used the Internet Archive’s Wayback Machine [18] to identify changes in smoking cessation content on the PM and QuitAssist websites from the late 1990s to the mid-2000s. For additional contextual information (e.g., evidence of implementation of a PM-funded program), we examined current and archived versions of the websites of several recipients of PM funds (KidsPeace, the Caron Foundation, and the Duke Center for Child and Family Policy), and reviewed the 2015 list of Altria (PM’s parent company) grantees (available on the Altria website). We also reviewed published scholarly articles assessing QuitAssist.

We analyzed documents and websites using an interpretive approach [19–22]. In this type of historical analysis, “the focus of attention is on meanings … Each document [is] reviewed carefully and the ‘taken for granted’ assumptions and viewpoints of the author[s] drawn out” [23]. Language is regarded as both conveying and constitutive of meanings [20].

Consistent with the analytic tradition within which we were working, we specified no pre-analytic conceptual schema [24–26]. To develop this interpretive account, the first author reviewed all documents and website material, and all authors reviewed selected key documents. Together, we compared and contrasted smoking cessation information on the PM and QuitAssist websites (obtained from the Wayback Machine), and information on youth smoking cessation programs that received funding from PM and on adult smoking cessation approaches considered by PM (obtained from tobacco industry documents and from the contextual sources outlined above), and took detailed notes. We relied upon iterative reviews of the documents and our notes to identify and evaluate common themes and “clusters of meaning” [23]. After reviewing material iteratively and identifying themes, we constructed a timeline of events, and assembled a case study [19, 27]. Approximately 100 documents were central to our case study, including those we cite and additional documents that helped provide context.

## Results

### Youth smoking cessation

In late 1997, when Mike Szymanczyk became PM’s chief executive officer (CEO), PM was facing many challenges. These included numerous state lawsuits to recover Medicaid costs for tobacco-caused disease, plummeting public opinion, and federal legislation that would have raised tobacco taxes, restricted tobacco advertising, set targets for reductions in youth smoking (and levied fines for failure to meet them), and brought tobacco under the purview of the Food and Drug Administration [28, 29]. Arguing that PM was “out of alignment” with society [30], Szymanczyk changed PM’s mission from a singular focus on being successful [31] to a focus on being “responsible, effective, and respected” [30]. Several months before the Master Settlement Agreement resolved most state lawsuits, PM created a Youth Smoking Prevention (YSP) Department, with an annual budget of $100 million [32], headed by Carolyn Levy, formerly marketing and sales senior vice president [33].

Although the department’s initial focus was on smoking prevention [34], smoking cessation was on its longer-term agenda. Looking ahead to 1999, a PM policy landscape assessment noted that “the issue of smoking by minors will continue to drive tobacco marketing restrictions and bans at the state and local levels … PM USA intends to be much more involved in youth smoking cessation efforts” [35]. Another internal explanation for PM’s interest in youth smoking cessation appeared to be to offer reassurance about the possibility and ease of quitting: “We have recognized the need to support youth smoking cessation programs since many kids who smoke believe it will be difficult for them to quit” [36, 37].

The YSP department did not attempt to develop its own youth cessation program, instead funding third parties to create new programs or implement existing programs. Several early partners were funded by PM despite reservations among YSP staff, and failed to achieve their goals (Table 1). The development of the department’s first long-term partnership—with the Caron Foundation (Caron; now Caron Treatment Centers), a Pennsylvania-based addiction treatment center–illustrated that the department took a similar approach to youth smoking cessation as it did with prevention, rejecting curricula with “anti-industry” themes [8].

**Table 1:** Examples of Philip Morris USA’s Youth Smoking Prevention Department’s early support for youth cessation programs, 1998–2002.

Date(s)	PM contribution	Recipient	Project	Initial YSP Department review	Outcome	YSP Department assessment
1998–2001	More than $1 million [38–41]	Ira Schwartz, University of Pennsylvania	Establish a “Children and Youth At-Risk Program” at the Center for the Study of Youth Policy at the University of Pennsylvania; one of its goals was to research youth smoking cessation [42, 43].	Not found.	Youth smoking cessation efforts consisted of investigating three “promising” programs & writing position paper “Preventing and Stopping Children from Smoking and Using Tobacco Products: Promising Policies and Strategies” [44–46].	Carolyn Levy: “I still believe that most of what Ira is doing is baloney and will continue to think that unless at the end they have something pretty amazing to report out.… wish that I had a crystal ball back in June of '98.” [47] Henry Fernandez: “disappointed with the … poor quality of work” [48].
2000–2001	$1.18 million [49–52]	Norman Hymowitz, University of Medicine and Dentistry of New Jersey & SmokEnders	Develop and evaluate “Take Charge” (renamed “N Control”) a youth smoking cessation program adapted from the SmokEnders adult program via a pilot test in three New York City high schools [49, 50, 52–55].	Identified possible flaw in the proposed design, an unrealistic timeline, and budget irregularities, and questioned whether PM wanted “to be associated with a program that possibly will have dubious success rates and limited applicability” [56, 57].	Project failed to meet any of its goals before depleting its funding [58, 59].	Not found.
2001	$100,000 [60]	KidsPeace (organization dedicated to helping kids overcome crises)	Create an online youth smoking cessation program at TeenCentral.net [59].	Questions regarding budget, efficacy of internet-based program, professionalism and expertise of KidsPeace staff [61–63].	No evidence on Internet Archive that a youth smoking cessation program was added to the TeenCentral.net website.	Not found.

In 2000, Levy began discussions with Caron to provide funding for a school-based youth smoking cessation program [64–68]. Caron chose the American Lung Association’s (ALA) “Not on Tobacco” (N-O-T) curriculum [69], and, after Levy reviewed it [67, 70, 71], the department agreed in early 2001 to provide Caron nearly $284,000 in funding [72]. Levy may have been interested in the ALA curriculum because public relations consultants Burson Marsteller had recommended it, noting that it was “one of the few programs that have been evaluated for efficacy” [73] (with preliminary research showing a 22.4% quit rate at 6 months) [74]. Even before PM gave Caron its first grant, the company sought to capitalize on the connection, asking Caron Vice President David Rosenkar to advocate for life-skills training, the tobacco industry’s preferred (and ineffective) approach to YSP [8], among Pennsylvania state legislators [75–77].

Caron experienced a setback In March 2001, when, upon being informed of the funding source, the ALA declined to approve “any program being provided in partnership with Philip Morris” [78] and the grant was suspended [79]. In discussions about next steps, Levy apparently expressed concern about how cessation programs portrayed the tobacco industry in curriculum materials [80, 81]. Caron responded by comparing tobacco-industry specific material in N-O-T and in two Community Intervention curricula: the Tobacco Awareness Program (TAP) and Tobacco Education Group (TEG) [80, 82]. Caron noted that the TAP facilitator’s guide “in no way bashes the tobacco industry” while TEG had some lessons with a “clearly … negative posture” towards the industry [80]. By contrast, N-O-T, despite having been approved by Levy, had the “most emphasis on the advertising of tobacco industries [sic] toward youth” [80]. Caron suggested that it could adopt TAP “without making any adjustments” and “adapt” TEG to eliminate the small number of “pieces that do have a negative posture towards the tobacco companies” [80]. Levy responded that she was not seeking to change curriculum content, and that her “sole objective continues to be finding and funding an effective youth smoking cessation program currently available in the marketplace” [81]. However, for unknown reasons, she asked Caron to continue “investigat[ing] programs other than TAP” [81].

In May 2001, after meeting with Levy, Caron agreed to submit a new funding proposal to implement “End Nicotine Dependence,” (END) a youth cessation program developed by Utah’s Department of Health [83]; it did not, at the time, contain anti-tobacco industry content [74, 84–86]. The following month, PM awarded Caron $300,700 to implement END in 53 eastern Pennsylvania schools [87, 88]. PM’s financial support for Caron’s youth cessation program continued until at least 2015 [89]. In 2006, there was a change in curriculum, with Caron developing its own program, Project CONNECT [90]. Although the curriculum includes a session on the health consequences of smoking, it does not appear to include critiques of the tobacco industry; it also incorporates life skills training, the preferred tobacco industry approach to YSP [8, 91]. We found no published evaluations of its effectiveness.

In 2005, PM expressed interest in creating a more “multi-faceted” youth smoking cessation program that included funding for scientific research to evaluate or develop more effective youth cessation programs and for implementing those found to be effective [92] However, we found no evidence that PM expanded its youth cessation program to incorporate those elements.

### Adult smoking cessation: PM’s “unique assets”

In 2000–2001, several task forces that had been created to explore PM’s new responsibility-focused mission recommended that the company create its own adult-focused smoking cessation programs or products [93–96]. To explore these ideas further, in 2002 PM created a Cessation Exploration Team (CET), composed of representatives of YSP, Worldwide Scientific Affairs, the legal department, Corporate Affairs, Philip Morris International, and several consulting firms [97, 98]. An early step was a review of quitting methods and cessation providers [99], and focus group research with smokers and former smokers to understand their knowledge of and attitudes toward cessation resources [99]. The CET concluded that while there was “a lot of [cessation] support in the marketplace,” it was not widely used [100], partly because providers had difficulty reaching smokers and failed to use the right tone (i.e., too “preachy”) [101]. Smokers perceived cessation services and aids as expensive, and found information hard to find and difficult to use [101]. Research on smoking cessation was also limited, focusing largely on pharmacological rather than psychosocial or sensory aspects of smoking [100].

The CET highlighted for senior management five “opportunities” offered by cessation support: “connecting with” consumers (nearly 70% of whom, they acknowledged, wanted to quit); societal alignment (a recently launched PM initiative to undertake “strategies and programs to meet society’s expectations of a responsible tobacco company”) [102]; leadership; partnership (presumably with individuals/organizations interested in smoking cessation); and business sustainability (although the CET did not specify how helping smokers stop using PM’s products advanced this goal) [98]. Nonetheless, the CET wondered if there was “an appropriate role for us to play” in adult cessation [97]. CET members concluded that despite PM’s “credibility challenges” and lack of cessation expertise, the company did have “unique assets” to offer, including knowledge of how to talk to adult smokers, access to smokers through marketing channels, scientific knowledge of reduced harm research, and financial resources [103].

#### How to talk to adult smokers

In pointing to PM’s knowledge of how to communicate with smokers, the CET seemed to suggest that PM’s marketing expertise could easily pivot from promoting cigarettes to discouraging their use. However, the CET handicapped itself at the outset by stating that its role was “not to convince adult smokers that they should quit.” Instead, its role was to “support an environment where if an adult smoker decides to quit smoking, he/she will be able to do so successfully” [98]. Early in the process of determining what that supportive role would be and how PM should communicate with smokers, CET consultants Lombardo Consulting Group (LCG) conducted 24 focus groups with former and current adult smokers and adult smokers trying to quit [99]. An initial stumbling block for participants was understanding *why* PM was offering smoking cessation help. Most assumed that PM was being forced to do so and questioned why a tobacco company would “want you to quit or want to help you quit?” [104]. Nonetheless, by the conclusion of the groups, there was near-universal agreement among participants that PM should offer some sort of cessation service [104]. The preferred name was “QuitAssist” [99, 104], and participants stressed that marketing would be “critical” to its success, with one participant stating that PM needed “to make quitting as appealing as they make smoking” [104].

First, however, LCG advised that PM’s motivation for supporting smoking cessation “ha[d] to be addressed” [104]. Otherwise, the public would be less receptive to whatever smoking cessation program PM chose to offer [104]. LCG convened additional focus groups to assess six potential explanations (crafted by PM) for PM’s motivation (Table 2). Most explanations touched upon multiple themes (e.g., both “business sustainability” and societal alignment). Several acknowledged public doubts, noting, for example, that “It doesn’t seem to make sense for a tobacco company that sells cigarettes to help adult smokers stop using them” [105]. Several explanations also failed to fully explain PM’s interest in smoking cessation. For example, PM might have the tools to help people quit, but why was it in their interest to do so? The CET did not settle on any of these explanations.

**Table 2:** Philip Morris USA focus group-tested explanations for its involvement in smoking cessation, 2003 [105].

Explanation	Example(s)
Societal alignment/business sustainability	• “It doesn’t seem to make sense for a tobacco company that sells cigarettes to help adult smokers stop using them. … [PM] USA is changing the way we do business. We are taking these actions to address what we understand to be the expectations of society of manufacturers that produce a product with serious health risks. We are also taking these actions to sustain the viability of our business.”
Societal alignment/business sustainability/harm reduction	• “Legislatures, states attorneys general, the public health community and society are all sending a message. They don’t like what they see as the practices of tobacco companies and they want to see cigarette manufacturers change some ways they do business and reduce the harm associated with their products … or face losing the right to be in business.”
Business impact/harm reduction	• “People will question our commitment to helping adult smokers who have decided to quit because of the potential impact they think it could have on the future of our business. It’s true … However … we believe that we will continue to have a successful business among adult smokers who want to smoke. … Reducing the harm associated with our product is an important expectation of society.”
Smokers gave permission/PM USA has tools to help people quit	• “We understand that people may not trust us or understand our motivation for helping adult smokers who want to quit. … We asked adult smokers if we could play a role in helping them if they decided to quit. Many said yes. … [W]e have the ability …. to connect them with expert quitting information and resources that can help them be more successful.”
PM USA has tools to help people quit	• “Philip Morris USA believes it can play a role in helping smokers who want to quit …. We have the ability to directly communicate with smokers.”
Meeting customer expectations/needs	• “We work to meet the preferences of consumers of our brands. . . by helping them find comprehensive information from a wide variety of expert sources about how to quit.”

#### Access to smokers through marketing channels

The CET considered relying on various marketing channels to communicate PM’s cessation message, including the web, print media, and PM’s database of 20 million smokers’ names, addresses, buying preferences and patterns, demographic information, and leisure activities [106]. For example, one proposal was simply to provide smoking cessation information to smokers by updating the quitting information on PM’s website and offering a printed version, and to use standard marketing practices to promote both (e.g., print advertisements and cigarette pack “onserts,” small brochures on the pack exterior) [97, 100, 101, 107]. An alternative marketing approach suggested by consultants Bain and Company (Bain) was to allow smoking cessation providers to access (for a fee) PM’s smokers database for targeted mailings [101]. Alternatively, if PM chose to fund the expansion of existing smoking cessation services (such as those offered by QuitNet or the ALA), it could include free access to PM’s smoker database to better target “want-to-quit” smokers [108].

#### Scientific knowledge of reduced harm

Another approach to smoking cessation that the CET considered throughout 2003 was to conduct or fund cessation research [100]. For example, PM could add cessation research to its scientists’ reduced harm research agenda in order to “enhance and innovate quitting method effectiveness” [99]. Alternatively, PM could offer funding to outside researchers through existing mechanisms, such as PM’s external research program [109] or the NIH-funded Transdisciplinary Tobacco Use Research Centers (a multi-university consortium studying tobacco use, addiction, and treatment) [110]. The group also debated creating an “expert cessation council” or “cessation advisory council” to conduct smoking cessation research or oversee the distribution of research funding to other institutions [100, 101].

Several proposals combined the provision of services with cessation science support.

For example, the CET considered a “partnership service” among cessation providers, scientific research centers, and PM that would “support enhanced service delivery and the advancement of proven-effective cessation methods for adult smokers who have decided to quit” [111]. Potential partners identified included QuitNet (for cessation services) and the University of California, San Francisco (for cessation science) [111]. (See Table 3 for a comprehensive list.) (It is unclear how many of these individuals/organizations PM ultimately approached. The CET acknowledged that there would likely be some “sensitivity” regarding an association with a tobacco company, and recommended that PM’s Worldwide Scientific Affairs department rely on its “scientific contacts” to informally assess outside interest in participating in a PM USA funded cessation science program) [112]. Alternatively, PM could establish a Cessation Treatment Scientific Research Center to help develop effective cessation programs and create a national network of cessation clinics to support those programs [113].

**Table 3:** Organizations and individuals identified by Philip Morris USA’s Cessation Exploration Team as potential smoking cessation program partners or “engagement candidates.” [97, 101, 107, 110, 111, 114, 115].

**Research Institutions**
Bowman-Gray University of Medicine (North Carolina)
University of California-San Francisco (Habit Abatement Clinic)
University of California-San Diego (Family and Preventive Medicine)
University of California-Berkeley (School of Public Health)
Kaiser Permanente
Mayo Clinic
Johns Hopkins (Quit Smoking Clinic)
Duke University
Transdisciplinary Tobacco Use Research Centers (Yale, Brown, University of Southern California, University of California-Irvine, University of Minnesota, University of Wisconsin, and University of Pennsylvania/Georgetown)
University of Michigan (Tobacco Research Network)
**Government Organizations**
Centers for Disease Control (CDC)
National Cancer Institute
Office of the Surgeon General
**Health Organizations**
American Lung Association
American Heart Association
American Cancer Society
**Commercial Organizations**
Weight Watchers
Walmart
**Smoking Cessation Service Providers/Substance Abuse Treatment Centers**
In-Control
Freedom from Smoking
Addiction Management Systems
SmokEnders
Smoke Stoppers
QuitNet
SmokeLess
Nicotine Anonymous
Free & Clear
Quit Smart
Global Health Cooperative
Pioneer Development
Robert Wood Johnson Foundation
Caron Foundation
Hazeldon Foundation/Betty Ford
Mountside
Progress Valley
La Hacienda Treatment Center
Mount Regis Center
Valley Hope Association
**Professional Organizations**
American Society of Preventive Medicine
American College of Epidemiology
**Pharmaceutical Companies**
GlaxoSmithKline
McNeil
Novartis
Pharmacia
**Engagement Candidates**
Rosemarie Henson, Director, CDC Office on Smoking and Health
Greg Connolly, Director, Massachusetts Tobacco Control Program
Joe Califano, Chairman of Center on Addiction and Substance Abuse, former Health Education and Welfare Secretary
David Kessler, Dean of Yale University School of Medicine, Former FDA Commissioner
Neil Benowitz, Chief of Division of Clinical Pharmacology and Experimental Therapeutics, University of California-San Francisco
Jed Rose, Chief of Nicotine Research Program, Duke University Medical Center
Mark McClellan, FDA Commissioner
Selected Attorneys General

The CET also discussed evaluating the effectiveness of any PMUSA cessation service in terms of public response to and awareness and use of the service [99] and quit rates via a clinical trial whose results could be published in a “scientific, peer-reviewed journal” [112, 116]. The intended evaluator was unclear–both internal and external evaluators were mentioned [99, 117].

#### Financial resources

The CET had noted from the outset that PM’s financial resources were an asset that would enhance its involvement in smoking cessation. A February 2003 document estimated the year 1 cost of a QuitAssist service that included a website, handbook, direct mail to PM smokers, telephone counseling, research, and point-of-sale advertising at $54.8 million, with the direct mailing accounting for a large portion of the cost ($19 million) [99]. Another option that Bain suggested could have the greatest impact on quit rates–a $25 coupon/rebate for FDA-approved cessation aids–would have added $11 million annually [101]. Alternatively, since low income smokers were less likely to quit and had lower success rates than other quitters, the CET considered establishing a cessation support fund (administered by a third party) to provide financial assistance for cessation services to low income smokers [118, 119]. Available documents did not indicate the projected cost of the fund.

### From proposals to action

#### Talking to smokers

PM ultimately chose to communicate its smoking cessation message to smokers via the web. On September 13, 2004, PM launched the QuitAssist website, describing it as “an information resource designed to connect smokers who have decided to quit to the wealth of quitting [sic] smoking resources offered by public health authorities and others”[120]. The site provided links to smoking cessation resources such as stop smoking services (e.g., QuitNet), publicly funded telephone quitlines, and stop smoking guides from organizations such as the American Cancer Society [121, 122]; it also featured short testimonials from successful quitters who described how they quit, the rewards of quitting, and how to cope with cravings and avoid weight gain [123]. A downloadable QuitAssist Guide, containing the website content, was available [123]. The site has maintained a continuous presence on the web since its launch.

The website did not, however, offer a clear explanation for PM’s involvement in smoking cessation. Instead, it noted the seeming contradiction and pointed out that “smoking causes serious diseases and is addictive. It can be difficult to quit … and many smokers who try … do not succeed” [123]. However, in other forums PM linked QuitAssist to its “mission of responsibility” and harm reduction goals:

Playing a role in supporting cessation aligns with our mission of responsibility and our goal to reduce the harm associated with our products. Other actions to support this goal include … playing a role in helping prevent youth smoking and working to develop products that may ultimately reduce the risk of disease and harm of smoking [124].

#### Access to smokers

PM advertised QuitAssist with a national print campaign [125] and a pack onsert, but no direct mailing to smokers in PM’s database [126, 127]. After three months, QuitAssist’s website had logged 110,000 visits and the company had distributed 80,000 booklets [127]. After one year, 680,000 website visits were logged [128]. In March 2005 PM began running 4 weeks of QuitAssist television advertisements [129].

#### Enhancing scientific knowledge

In June 2004, PM awarded Duke University professor Jed Rose $15 million to establish the Duke Center for Nicotine and Smoking Cessation Research [130]. (By 2012 PM had provided over $37 million to the Center) [131]. Its focus was developing, evaluating, and distributing “improved smoking cessation methods” [132]. The contract required Duke to acknowledge PM’s financial support, but did not specify particular research topics [130].

Although we identified descriptions of two studies undertaken by Duke University investigators to evaluate QuitAssist’s effectiveness [133, 134], we found no PM documents or peer-reviewed publications that reported results, and the principal investigator of one study confirmed the lack of published data (personal communication with Rick Hoyle April 2016). However, independent research has found that, compared to other smoking cessation websites, smokers and former smokers who visited the QuitAssist website were more likely to indicate that the site did not help them quit or to say that “it seemed to make things worse” [10]. PM’s QuitAssist messaging also proved to be problematic in independent research. Smokers who watched a QuitAssist ad were more likely to smoke afterwards than those who viewed either an American Legacy Foundation “truth” antismoking ad or a public service ad unrelated to smoking. This “boomerang” effect was attributed to QuitAssist’s repeated visual and auditory references to smoking and cigarettes [12]. Similarly, research exploring the relationship between aided awareness of smoking cessation media campaigns and cessation over time found that aided awareness of QuitAssist ads was associated with decreased confidence in quitting and lower likelihood of a quit attempt [11].

#### Financial resources

PM devoted $17 million to QuitAssist in 2003–2004 [32]. (It is unclear if that figure included advertising). This was much less than the figures outlined in CET proposals that had included telephone counseling and a $25 coupon for cessation aids, and much less than PM devoted in 2003 to marketing its products (Table 4). Once the website was launched, ongoing publicity on television, radio, and the web accounted for the majority of its costs [135]. In 2005, PM planned to spend 26.5% of its corporate responsibility budget on cessation support advertising ($24.4 million), and 32.5% on YSP advertising ($29.9 million) [136]. That same year, a “responsibility leadership team” noted (without elaboration) an opportunity for PM to play a role in “reducing out of pocket cost of cessation treatment” [137]; however, an unidentified commenter noted that “in light of new business endeavors, we are not sure this is an appropriate business ‘opportunity’” [137]. It is unclear if the commenter was indicating that the cost of the new endeavors precluded additional cessation spending, or that such spending was incompatible with the content of the new endeavors. At the time, PM was preparing to test market Marlboro Ultrasmooth, a “potentially reduced exposure product” with a new filter that, in theory, reduced exposure to smoke toxicants [138, 139].

**Table 4:** Philip Morris USA expenditures on tobacco marketing and adult smoking cessation programs, 2003–2004.

Tobacco marketing (2003)	QuitAssist website (2003–2004)	Proposed smoking cessation program, including website, telephone counseling, & direct mail to smokers (2003)	Proposed smoking cessation program, including website, telephone counseling, direct mail to smokers, & $25 coupon for cessation aids (2003)
$8.3 billion [140]	$17 million [32]	$54.8 million [99]	$65.8 million [101]

#### Engaging with public health

PM sent letters to 21 organizations and 50 states whose services were mentioned in the QuitAssist guide [127], noting that the guide contained a disclaimer: “references to organizations do not indicate or imply their endorsement, support or approval of either the contents of the resource or the policies or positions of Philip Morris USA” [120]. It also sent letters to 8 additional (unnamed) organizations “seeking engagement” [127]. It is unknown if PM was successful in engaging with these organizations; however, a member of the YSP department reported to PM’s litigation team in December 2004 that the QuitAssist launch served as an opportunity to engage with the American Legacy Foundation, the Academy for Educational Development, and the Virginia Tobacco Settlement Foundation on the issue of youth cessation [127].

## Discussion

Our study sheds light on PM’s interest in and approach to smoking cessation. The same forces that propelled its involvement in youth smoking prevention were at play when it came to youth smoking cessation: public and policymaker interest in implementing tobacco control policies to address growing concerns about the problem of youth smoking. PM’s prevention and cessation programs effectively suggested that such policies–which threatened its interests—were unnecessary.

PM also reportedly hoped that its support of youth cessation would reassure youth who believed that smoking cessation was difficult. Smoking cessation is, in fact, difficult for youth [141]; however, studies indicate that adolescent smokers holding this belief are in the minority. Instead, most suffer from an “optimistic bias” when it comes to smoking cessation, believing that quitting will be easy to accomplish once they have finished “experimenting” with smoking [142–144]. Reinforcing this bias–whether through a PM-supported youth cessation program or other means–could prolong or encourage youth smoking, an outcome that would align with PM’s long term business interests but conflict with its stated goal of helping to reduce youth smoking and its goal of appearing responsible.

In entering the youth smoking cessation arena, the YSP department had a string of early failures, a likely consequence in several instances of setting aside initial reservations about grantees. Levy may have felt compelled to do so by a pressing need to demonstrate that the newly-established and well-funded department was doing *something* about the problem of youth smoking. This apparent urgency to proceed may also explain why Levy only raised questions about youth cessation curricula with anti-industry themes AFTER she had already approved a curriculum that included those same themes. Once the department began working with Caron, its approach to youth smoking cessation was similar to its approach to youth smoking prevention: despite claims to be only interested in effectiveness, support only those programs that are not critical of the tobacco industry [8]. Research has shown that media campaigns with counter-industry themes are highly effective in changing attitudes towards tobacco and reducing smoking initiation and smoking prevalence among youth [145–149].

One positive element of the PM/Caron story was the ALA’s refusal to allow a PM-funded organization to license its cessation curriculum. This underscores the importance of tobacco control organizations retaining ownership of such materials. Without such control, there is no guarantee that the curriculum will be delivered as intended, or that corporate funders will not seek to benefit from an association, however tenuous, with a respected public health organization.

PM’s interest in adult smoking cessation was harder to understand than its interest in youth smoking cessation. Indeed, the company itself had a difficult time articulating it: the CET offered a variety of rationales to senior management and focus group-tested additional explanations before settling on an assertion that its support for smoking cessation was consistent with its broader interest in harm reduction. CET documents suggest that PM’s involvement in adult smoking cessation was not, at the outset, intended as an empty public relations gesture or an attempt to undermine cessation: CET members conducted extensive research on smoking cessation and proposed a wide range of cessation approaches and potential partners. Nonetheless, the final product was markedly unambitious compared to many of the alternatives considered, consisting mainly of educational content taken from and links to other websites. The boldest elements—those that might have had a larger impact on cessation rates than a website—were missing: telephone counseling, partnerships with existing smoking cessation providers, and targeted mailings to smokers using PM’s smoker database. A particularly notable absence was the lack of financial support to help smokers purchase NRT, the very action that consultants Bain and Company had argued would allow PM to have the largest impact on quit rates. The planned evaluation of QuitAssist’s effectiveness in actually reducing smoking was also missing (at least from public view).

The transformation of an ambitious plan into a mundane final product is a recurring theme with PM’s corporate responsibility efforts [148, 150, 151]. One explanation may be that this is a natural process that organizations undergo, forced to compromise as they encounter obstacles in the process of translating bold ideas into concrete actions. For QuitAssist, one likely obstacle that was minimally discussed was the low likelihood of various public health organizations or academics partnering with a tobacco company on this endeavor. The only university partner to emerge, Duke University, already had ties to PM as well as longstanding tobacco connections dating to the 1920s [131]. The amount of money the company was willing to commit to adult smoking cessation support may also have played a role in the underwhelming final product–approximately $17 million in its first year, about one-third of the cost of the more comprehensive proposals the CET considered. The CET’s narrow view of PM’s role in cessation–supporting but not advocating it–may have also led to the rejection of certain proposals, such as direct mail to smokers in PM’s database, that could more easily cross over into the realm of advocating cessation.

A more fundamental problem, however, was the conflict of interest that PM faced in supporting or creating *any* type of smoking cessation program. PM’s primary fiduciary obligation to shareholders is to maximize profits by increasing sales; why, then, would the company want to help smokers stop using its products? Its ultimate explanation, an interest in harm reduction, appears sensible only by ignoring that the company continues to aggressively market deadly and addictive products, enhance cigarettes’ addictive properties, [152, 153] and oppose or undermine tobacco control policies [154–156].

PM may not have intended for QuitAssist to undermine cessation, but independent researchers’ finding to that effect [10–12] suggests that QuitAssist cannot be dismissed simply as a half-hearted attempt by PM to appear responsible. It has the potential to inflict real harm by competing with more effective programs [10]. Another source of harm is the role that seemingly responsible corporate initiatives like QuitAssist play in maintaining a tobacco-favorable policy environment. As we have seen with tobacco industry sponsored YSP programs, these initiatives undermine tobacco control by providing policymakers with reasons to engage with the industry [157–159], and by suggesting that strong regulation of a “responsible” industry is unnecessary [6, 7]. QuitAssist was designed to provide engagement opportunities for PM with a variety of public health and government officials, while PM’s support for youth smoking cessation was intended to blunt policymaker interest in marketing restrictions.

If PM truly wanted to support rather than undermine cessation, there are many low or no-cost steps it could take, including eliminating direct mail and other types of promotions to customers in its database, dropping legal and other challenges to public policies that discourage smoking—such as better health warnings, raising the age of tobacco purchase to 21 [160, 161] and higher tobacco taxes [162, 163]—and removing the QuitAssist website.

Our findings bear similarities to the corporate social responsibility strategies of other industries. The alcohol industry’s “responsible drinking” programs (which have a longer history than PM’s cessation programs) have been found to promote drinking and the industry’s public relations interests and policy preferences [164–166]. Likewise, soda companies’ youth-focused responsibility campaigns aim to increase sales and prevent regulation [167]. Given the harms associated with all three industries, it is perhaps not surprising that they would resort to similar defensive strategies to protect their profits.

Our study has limitations. Due to the size of the document databases, we may not have retrieved every relevant document. Some may have been destroyed or concealed by tobacco companies [168]; others may have never been obtained in the legal discovery process, including, for example, PM-sponsored research on QuitAssist. In addition, because many of the CET-related documents we found were slide presentations lacking extensive detail, our knowledge of why certain ideas were later abandoned is limited.

## Conclusions

Tobacco company involvement in smoking cessation represents a threat not only to individual smokers, whose quitting efforts may be undermined by ineffective programs, but also to public health more broadly, by positioning the industry as part of the solution to the tobacco epidemic. Making sense of tobacco companies’ cessation initiatives may be easier when they stop selling cigarettes, as Philip Morris International has claimed it intends to do [169]. Until then, such efforts should be regarded skeptically.
